# Beer Production With Umbu (*Spondias tuberosa*) Using Conventional and Nonconventional Yeast

**DOI:** 10.1111/1750-3841.70875

**Published:** 2026-01-18

**Authors:** Diego Pádua de Almeida, Hygor Lendell Silva de Souza, Ana Luiza Dozol, Mariana Moysés Delorme, Vanessa Naciuk Castelo‐Branco, Adriene Ribeiro Lima, Alexandre Soares dos Santos, Nadia Nara Batista, Rosane Freitas Schwan, Gustavo Molina, Cintia Lacerda Ramos

**Affiliations:** ^1^ Institute of Science and Technology Federal University of Jequitinhonha and Mucuri Valley Diamantina Minas Gerais Brazil; ^2^ Phamarcy School Fluminense Federal University Niteroi Rio de Janeiro Brazil; ^3^ Basic Sciences Department Federal University of Jequitinhonha and Mucuri Valley Diamantina Minas Gerais Brazil; ^4^ Biology Department Federal University of Lavras Minas Gerais Brazil

**Keywords:** antioxidant activity, bioactive compounds, craft beer, phenolics, *Pichia kluyveri*

## Abstract

This study evaluated the fermentation performance and bioactive properties of beers produced by *Pichia kluyveri* L131 and commercial *Saccharomyces pastorianus*, individually and in coculture, in wort enriched with umbu pulp. Fermentation profiles were assessed over 12 days, focusing on yeast population dynamics, carbohydrate consumption, and metabolite production. Antioxidant and antimicrobial activities and volatile and bioactive compounds were evaluated in wort and fermented assays. While *P. kluyveri* demonstrated lower maltose metabolism and ethanol production (6.07 g/L), it produced higher fruity and floral ester concentrations, contributing to a unique sensory profile. *P. kluyveri* exhibited beers with increased antioxidant activity and phenolic compound retention compared to those produced with *S. pastorianus*. Cocultivation preserved key characteristics of both yeasts, mainly regarding volatile compounds. Antimicrobial activity varied across assays, inhibiting *L. monocytogenes* and *S. aureus* in *P. kluyveri* beers. These findings show *P. kluyveri's* potential for low‐alcohol beer production, enhanced flavor complexity, and bioactive properties.

## 1 Introduction

Beer is an alcoholic beverage widely consumed worldwide and fermented mainly by *Saccharomyces* yeast. Studies have been performed evaluating yeasts, both *Saccharomyces* and non‐*Saccharomyces*, to identify novel strains capable of producing fermented alcoholic beverages with distinct sensory characteristics (Chua et al. 2018; Tanguler and Sener 2022; Francesca et al. 2023). Non‐*Saccharomyces* yeasts, such as species of *Pichia, Saccharomycodes, Zygosaccharomyces, Hanseniaspora*, and *Torulaspora*, have been studied for their potential use as starter cultures in beer production (Basso et al. 2016; Adamenko et al. 2020; Canonico et al. 2021; Matraxia et al. 2021; Durga Prasad et al. 2022; Miguel et al. 2022; Romero‐Rodríguez et al. 2022; Canonico et al. 2023; Hinojosa‐Avila et al. 2024; Hlangwani et al. 2024). The yeast species *Lachancea thermotolerans* can produce lactic acid, allowing the creation of sour beers without adding lactic bacteria. This fermentation process is shorter and has positively impacted the flavor and aroma of the beer (Francesca et al. 2023).

Studies have shown that *Pichia kluyveri* strains can increase the levels of acetate esters, giving beer fruity characteristics (Holt et al. 2018; Vicente et al. 2021; Methner et al. 2022; Piraine et al. 2023). Non‐*saccharomyces* yeast, including *P. kluyveri*, is a producer of enzymes, especially hydrolases such as glycosidase, capable of releasing aroma precursors or aromatic substances. However, they perform limited alcoholic fermentation, producing lower alcohol contents than the *Saccharomyces* yeast species. Thus, it represents a promising strategy for low‐alcohol fermented beverages or for addition as a coculture with commercial yeasts, acting as flavoring agents (Sadineni et al. 2012; Ruiz et al. 2018; Matraxia et al. 2021).

Another interesting aspect that has been evaluated is the addition of fruit pulps to produce distinct beers (Patraşcu et al. 2018). Traditional beer producers have adapted their methods to include fruits, producing new varieties like sour fruit beers. A notable example is Belgian lambic beer, made with a combination of barley malt and non‐malted wheat, which receives the addition of cherries or raspberries, undergoing a spontaneous fermentation process. These beers have become popular due to their robust fruity flavor and refreshing properties derived from their pleasant acidity (Spitaels et al. 2017; Gorzelany et al. 2022).

The use of fruits to produce fermented beverages such as beer has demonstrated great potential in adding sensory and nutritional value (Aquilani et al. 2015; Zapata et al. 2019; Gorzelany et al. 2022). Fruity beers are produced by adding fruit pulps, extracts, or flavorings. Adding extracts or pulp fruits to beer can increase the concentrations of their bioactive compounds, such as compounds with antioxidant properties. Extracts and pulps obtained from fruits are recognized for their high levels of phenolic compounds, which have shown potential in terms of hypotensive, antibiotic, or anti‐inflammatory properties (Kucharska et al. 2015; Ducruet et al. 2017; Kawa‐Rygielska et al. 2018; 2019; Baigts‐Allende et al. 2021).

Umbu (*Spondias tuberosa)* is a native fruit of the Brazilian semiarid region (N. L. Rodrigues et al. 2024) and has received special attention from researchers due to its high contents of bioactive compounds and antioxidant properties (Ribeiro et al. 2019; Júnior et al. 2021; Macêdo et al. 2023). The presence of vitamins, including riboflavin, nicotinamide, pantothenic acid, vitamin C, minerals including phosphorus, potassium, calcium, magnesium, iron, and bioactive compounds such as anthocyanins, flavonoids, carotenoids, and chlorophyll, has been described in umbu fruits (Rufino et al. 2010; Schiassi et al. 2018; Ribeiro et al. 2019, Assis et al. 2020; N. L. Rodrigues et al. 2024). However, these fruits remain underexplored and show potential for their application in developing food products such as fruit beers.

The objective of this work was to evaluate the chemical, antioxidant, and antimicrobial properties of beers produced by unconventional yeast *Pichia kluyveri* L131 in single culture and coculture with commercial yeast *Saccharomyces pastorianus*, in wort with the addition of umbu pulp (*S. tuberosa*).

## 2 Material and Methods

### 2.1 Chemicals

Commercial standards, including glucose, fructose, maltose, ethanol, glycerol, acetic acid, succinic acid, gallic acid, 5‐caffeoylquinic acid (5‐CQA), caffeic acid, catechin, 4‐hydroxybenzoic acid, 3,4‐dihydroxybenzoic acid, ferulic acid, sinapic acid, *p*‐coumaric acid, and caffeine, were obtained from Sigma‐Aldrich Chemical Co. (São Paulo, Brazil). All solvents used were chromatographic grade and purchased from Tedia (Rio de Janeiro, Brazil). Alkane standard mixture for GC‐MS analysis and the Folin–Ciocalteu reagents, 2,2‐diphenyl‐1‐picrylhydrazyl (DPPH), and 2,4,6‐tripyridyl‐s‐triazine (TPTZ) were obtained from Sigma‐Aldrich (St. Louis, MO, USA).

### 2.2 Yeast Propagation

The yeast *Pichia kluyveri* L131, isolated from kombucha and belonging to the culture collection of the Microbiology Laboratory of UFVJM (Diamantina‐MG), was previously evaluated for beer production (Silva de Souza et al. 2025), and the commercial yeast *S. pastorianus* (Saflager s‐23, Fermentis) was used. The *S. pastorianus* Saflager s‐23 was used due to its relatively low production of esters and higher alcohols, as well as its suitability for fermentations conducted at temperatures up to 22°C (according to manufacturing instructions). For fermentation, the dry commercial yeast (10% w/v) was rehydrated in sterile water at 22°C for 30 min. Then, the inoculum was adjusted to reach a final concentration of 1 × 10^8^ cells/mL to ensure consistent dominance of the inoculated strain, and added to the brewery wort (according to manufacturing instructions). Higher inoculation levels have been shown to accelerate fermentation, and non‐*Saccharomyces* strains often require higher initial cell densities to express their metabolic potential within laboratory fermentation scales.

The non‐*Saccharomyces* yeasts were propagated in 10 mL of YEPG broth (1% yeast extract, 2% soybean peptone, and 2% glucose) at 30°C for 24 h. Then, the yeasts were inoculated in 90 mL of YEPG medium for another 24 h at the same temperature. After growth, the yeasts were centrifuged in a Romerlab SL‐706 Refrigerated Benchtop Centrifuge (2058 ×* g* for 10 min), washed with sterile distilled water to remove the culture medium, and then inoculated in 100 mL of brewery wort (Vrînceanu et al. 2025). Three trials were performed, one containing only *P. kluyveri* L131, another containing only commercial *S. pastorianus*, and a third trial inoculated with both yeasts. In all trials, inoculation was performed at a concentration of 1 × 10^8^ cells/mL. In the third trial, which involved co‐fermentation of the yeasts in equal proportions (1:1) (Huang et al. 2024; van Rijswijck et al. 2017), the total concentration was adjusted to 1 × 10^8^ cells/mL.

### 2.3 Preparation of Brewing Wort and Beer Fermentation

The brewing wort was prepared using 6% powdered malt extract (Dry Brew, Liotécnica, São Paulo, Brazil) diluted in potable water and heated to 80°C for 15 min. This malt extract is a concentrated powder made from malted barley, serving as an easy‐to‐use ingredient in beer brewing and replacing traditional malt. Then, 2.4 g of 30% iso‐α‐acid hops (Prodooze) were added. The wort was filtered to separate the solid phase and cooled to 20°C for the addition of umbu pulp and yeast. The sterility of the medium was confirmed by the absence of colony growth after plating on YEPG and PCA (Plate Counting Agar, Himedia) media. Fermentations were carried out in 150 mL flasks containing 75 mL of wort together with 25 mL of pasteurized commercial umbu pulp (obtained from Cooperativa Grande Sertão, Montes Claros, Brazil, pH 2.6; 10°Brix; 8.9% total sugar; 5.7% fermentable sugar; 0.6% protein; 0.2% lipids; 2.7% fibers), inoculated with yeasts (1 × 10^8^ cells/mL), and incubated for 12 days at 20°C. The wort had a pH of 5.1 and an original extract of 12.2°Plato. The fermentations were performed in triplicate and repeated twice.

During fermentation, weight loss analyses were performed to evaluate CO_2_ release. Analysis of apparent extract, cell count in a Neubauer chamber, and evaluations of carbohydrates (glucose and maltose), glycerol, ethanol, and acetic acid were carried out at 0, 2, 4, 6, 8, and 12 days of fermentation. The fermentation ended when no further modification in the concentration of evaluated carbohydrates was observed. The volatile compounds, total phenolics, antioxidant properties (DPPH and Ferric Reducing‐antioxidant Power [FRAP]), profile of phenolic compounds, and antimicrobial activity were evaluated at 0 and 12 days of fermentation.

### 2.4 Carbohydrates, Alcohols, and Organic Acid Analysis

Glucose, fructose, maltose, ethanol, glycerol, acetic acid, and succinic acid during fermentation were analyzed by HPLC according to the methodology adapted from Matos et al. (2018). A Shimadzu Prominence UFLC 20A system was used, equipped with a Rezex ROA‐Shodex column (300 × 7.8 mm) maintained at 60°C and using H_2_SO_4_ 0.0025 mol L^−1^ as eluent at 0.6 mL/min. The wort and samples were injected automatically in volumes of 5 µL. Refractive index detection determined carbohydrates and alcohol. A UV detector was used to detect organic acids. External standards and calibration curves were used for qualitative and quantitative determinations. The analysis was performed in triplicate.

### 2.5 Volatile Compounds Analysis by Gas Chromatography

The volatile compounds present in the wort and after 12 days of fermentation were extracted from the headspace of the samples by the solid‐phase microextraction technique using a Divinylbenzene/Carboxen/Polydimethylsiloxane (DVB/CAR/PDMS) SPME fiber (Supelco Co., USA). Samples (2 mL) were added to hermetically sealed 20 mL vials containing NaCl at 4% (m v^−^ **^1^**) and heated for 10 min at 50°C. The volatile compounds were extracted by inserting the SPME fiber at the top of the vial (50°C for 20 min). The desorption was performed by placing the fiber in the injection port of the gas chromatograph coupled to a mass spectrometer (GC–MS) (QP‐PLUS‐2010, Shimadzu, Japan) equipped with an Rtx‐5MS column (30 m × 0.25 mm × 0.25 µm). The column temperature was programmed as follows: the oven temperature was maintained at 50°C for 5 min, then raised to 200°C in increments of 3°C/min, and then kept at 200°C for 15 min. The injector temperature was 230°C and remained for 3 min. The ion source temperature was 200°C, the interface temperature was 200°C, and the solvent cut‐off time was 2 min. Helium (He) was a carrier gas maintained at a flow rate of 1.2 mL/min (Evangelista et al. 2014). The volatile compounds were identified by comparing their retention time with the C7–C40 saturated alkanes standard and the Shimadzu database (Wiley 7, NIST 62, NIST 12, NIST 05 s, NIST 05). Linear retention indexes (LRI) of the compounds were calculated using a series of alkanes (C7–C40) injected in the same chromatographic conditions. The analysis was performed in duplicate.

### 2.6 Total Phenolics Determination

The total phenolic content was determined by the Folin‐Ciocalteu assay using gallic acid as a standard, as described by de Miranda et al. (2023). A sample volume of 0.5 mL was diluted with 2.5 mL of the Folin‐Ciocalteu reagent (diluted 1:10 with de‐ionized water, *v/v*) and was neutralized with 2 mL of sodium carbonate solution (4.0% w/v). The reaction mixture was kept in the dark for 2 h and then absorbance was measured by a UV–VIS 2600 spectrophotometer (Shimadzu, Kyoto, Japan) at 740 nm. The total content of phenolic compounds was expressed as µg gallic acid equivalent per mL of sample (µg GAE/mL) and calculated based on the straight‐line equation generated from the calibration curve at concentrations 10, 20, 30, 40, 60, and 80 µg/mL. The analysis was performed in triplicate.

### 2.7 DPPH Antioxidant Assay

Antioxidant activity was assessed using the DPPH free Radical Scavenging Activity assay. DPPH (1,1‐diphenyl‐2‐picrylhydrazyl) free radical scavenging activity measurements were performed with modifications as described by Silva et al. (2021). In an amber vial, volumes of 3.9 mL of DPPH solutions in ethanol (0.06 mM) were added to 0.1 mL of each sample. The mixture was stirred for 30 s using a mixer (Kasvi, Brazil) and kept in the dark. After 30 min, a reading of 517 nm was recorded using a UV–VIS 2600 spectrophotometer (Shimadzu, Kyoto, Japan). The analysis was performed in triplicate. A blank experiment was performed using the same procedure without the beverage samples, and the absorbance was measured. The lower absorbance indicated free radical scavenging activity. The free radical‐scavenging activity of each beverage was then calculated as the percent inhibition according to the following equation:

$$\%\;\mathrm{inhibition}=100\left[A\left(\mathrm{blank}\right)-A\left(\mathrm{sample}\right)\right]/A\left(\mathrm{blank}\right)$$

### 2.8 FRAP Assay

The Ferric Reducing Antioxidant Power (FRAP) method was employed to assess the iron‐reducing capacity of the wort and beer samples. The methodology followed was based on the procedure described by Pulido et al. (2000), with some modifications. Three dilutions of the samples (1:5, 1:10, and 1:20) were prepared in triplicate test tubes. In a dark environment, 0.03 mL of each dilution was transferred to tubes containing 0.09 mL of distilled water and 0.9 mL of the FRAP reagent. The FRAP reagent contained 2.5 mL of a 10 mmol/L TPTZ solution in 40 mmol/L HCl plus 2.5 mL of 20 mmol/L FeCl_3_‚6H_2_O and 25 mL of 0.3 mol/L acetate buffer, pH 3.6. After homogenization, the samples were incubated in a water bath at 37°C for 30 min and kept in the dark to prevent photochemical degradation. Absorbance readings were then taken at 595 nm using a UV–VIS 2600 spectrophotometer (Shimadzu, Kyoto, Japan). Antioxidant capacity was quantified using a ferrous sulfate standard curve (500 µM to 1500 µM). The results were expressed in mM ferrous sulfate equivalent per liter (mM FeSO_4_/L), calculated from the absorbance values of the different dilutions.

### 2.9 Analysis of Phenolic Compounds

Phenolic compounds were analyzed by high‐performance liquid chromatography (HPLC) following the method of Inada et al. (2015). The sample was prepared as described by Moura‐Nunes et al. (2016) with adaptations. Briefly, 2 mL of the sample and 200 µL of each Carrez solution were added to a 5 mL volumetric flask, and the volume was completed with distilled water. After homogenization and resting for 15 min, the mixture was filtered through Whatman no. 1 filter paper, freeze‐dried, and reconstituted with 1.2 mL ultrapure water. Before HPLC injection, samples were filtered through a cellulose ester membrane (0.45 µm).

HPLC analysis was conducted using Shimadzu equipment (Tokyo, Japan), including an LC‐20AT quaternary pump, SPD‐M20A diode array detector (DAD), CBM‐20A control system, DGU‐20A5 degasser, and SIL‐20AC automatic injector. Chromatographic separation of the compounds was achieved using a reverse‐phase C18 column (250 × 4.6 mm, 5 µm, Kromasil100197, Bohus, 198, Sweden) and a gradient mobile phase consisting of 0.3% formic acid (A), methanol (B), and acetonitrile (C) at a flow rate of 1.0 mL/min. Eluent C concentration was kept constant at 1% during analysis. Before injection, the column was equilibrated with 18% B. The gradient elution program was as follows: 18% (B) at 1 min, 20% (B) at 18 min, 43% (B) at 30 min, 85% (B) at 40 min, followed by 10 min of re‐equilibration, for a total analysis cycle time of 50 min. Detection was carried out at wavelengths ranging from 254 to 370 nm. Quantification was performed by external standardization with a calibration curve ranging from 0.25 to 20 µg/mL. Chromatographic data were processed using LC Solution software (Shimadzu, version 1.25). Results are expressed as µg/mL, with analyses conducted in triplicate.

### 2.10 Antibacterial Activity

Bacterial strains of food importance, *Listeria monocytogenes* (CLIST2194), Enteropathogenic *Escherichia coli* (EPEC) INCQS 00185 (CDC 0127a), Enterohemorrhagic *E. coli* (EHEC) O157:H7 (RJ 691/1), *Staphylococcus aureus* (ATCC 13565), *Salmonella enterica* subsp. *enterica* serotype Typhimurium (*Salmonella* Typhimurium) (IOC 190), and *S. enterica* subsp. *enterica* serotype Enteritidis (*Salmonella* Enteritidis) (8806/9), obtained from the Collection of Reference Microorganisms in Health Surveillance (CMRVS) of the National Institute for Quality Control in Health (INCQS) of FIOCRUZ, were used to evaluate antibacterial activity of the wort and beer samples. The strains frozen at −20°C were reactivated on Soy Tryptone Agar (TSA) plates (Kasvi, Brazil) and incubated at 35°C for 24 h.

Antibacterial activity was evaluated using a broth microdilution method. The minimum inhibitory concentration (MIC) and the minimum bactericidal concentration (MBC), the lowest concentration of a substance capable of killing a particular strain of bacteria, were determined. The analyses were performed in triplicate in 96‐well microplates (CLSI 2012). Beer samples, both with pH neutralized with NaOH 1 M and non‐neutralized, were used to evaluate antimicrobial activity.

Isolated colonies were transferred from the TSA plates to tubes containing tryptone soy broth (TSB) (Kasvi, Brazil) and incubated under stirring at 35°C for 24 ± 2 h. The inoculum was standardized until it reached a turbidity equivalent to 0.5 on the McFarland scale (10^8^ CFU/mL).

Serial dilutions were prepared by adding 100 µL of a sample to 100 µL of TSB broth. The samples' concentrations ranged from 500 to 1 µL/mL. Subsequently, 100 µL of the standardized bacterial culture was inoculated into all wells except the negative control wells (TSB broth). The final volume of each well was 200 µL, and after micropipetting, the microplates were capped and incubated at 35°C for 24 ± 2 h.

All wells were visually observed once the incubation period was over to check for microbial growth (CLSI 2012; de Miranda et al. 2023). Turbidity or the presence of precipitate was indicative of growth. MBC was determined from wells that did not show turbidity, indicative of bacterial inhibition. An aliquot of the contents of the wells, which showed no growth, was plated with TSA and incubated at 35°C for 24 ± 2 h to determine the presence or absence of microbial colonies. MBC was considered the lowest concentration when no colonies in TSA were observed. Analyses were also carried out, as described above, to determine whether the ethanol in the beers contributed to their antimicrobial activity, using three ethanol solutions with concentrations corresponding to the beers. This procedure assessed ethanol's influence on the beer's antibacterial activity. All tests were performed with the control Chloramphenicol (CLO) at concentrations ranging from 100 to 0.2 µg/mL, TSB broth, and TSB broth with added bacterial inoculum.

### 2.11 Statistical Analysis

The fermentations were performed in triplicate and repeated twice. The analyses were performed in triplicate. The values are expressed as means ± standard deviation of the results. Analysis of variance (ANOVA) followed by Tukey's post hoc test was used to compare beverages at different fermentation times. Statistical analyses were performed using Jamovi software (version 2.3.28). *p* < 0.05 were considered significant. Heatmap was built using heatmapper (http://www.heatmapper.ca), with average linkage as a clustering method and Euclidian distance.

## 3 Results and Discussion

### 3.1 Yeast Population, Apparent Extract, and CO_2_ Production During Fermentation

**FIGURE 1:**
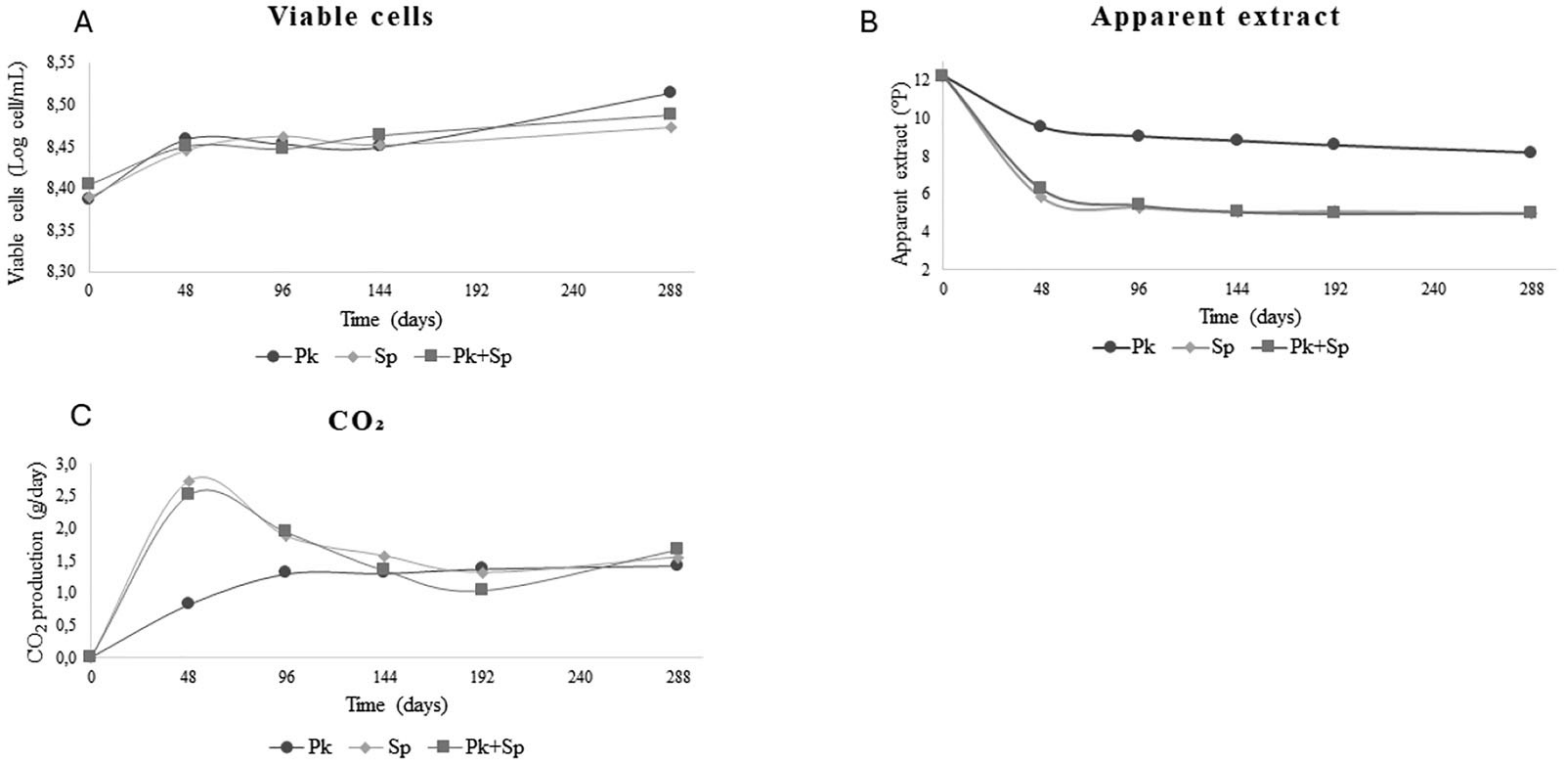
Viable cells (Log cell/mL) (A), Apparent extract (°P) (B), and CO_2_ (C) production (g/day) in single and cocultivation during 12 days of fermentation using *P. kluyveri* L131 (Pk), commercial *S. pastorianus* (Sp), and *P. kluyveri* L131 plus commercial *S. pastorianus* (Pk+Sp).

The yeasts *P. kluyveri* and *S. pastorianus* were evaluated as single cultures and cocultures for beer production in wort with umbu pulp. *P. kluyveri* L131 has been previously evaluated for beer fermentation and has demonstrated potential to produce low alcoholic beer (Silva de Souza et al. 2025). Fermentations were evaluated over 12 days, and yeast population, apparent extract (in °P), and CO_2_ releasing are shown in Figure 1. The yeast populations remained above 8 Log cell/mL throughout the fermentation in all trials (Figure 1A). There was no significant difference (*p* > 0.05) between the assays, suggesting adaptation and stability of the two yeasts in the brewing wort with umbu pulp throughout the fermentation period.

In beer production, reduced wort density indicates that the yeast is metabolizing fermentable sugars (Laureys et al. 2020). Original extract values were 12,2°P and, throughout the 12 days of fermentation, a consistent decrease in apparent extract was observed in all trials, reaching values around 5°P for both assays containing *S. pastorianus* (single and cocultivation) and 8.2°P for the *P. kluyveri* in a single culture, as shown in Figure 1B. The assay containing the unconventional yeast *P. kluyveri* L131 in a single culture showed the minor (*p* < 0.05) reduction in the *p* value. Regarding CO_2_ production, the assay containing only *P. kuyveri* L131 showed lower (*p* < 0.05) CO_2_ losses in the first 4 days compared to the other trials (Figure 1C). These data suggest a later fermentation when compared to the assays containing *S. pastorianus* (single or co‐fermentation).

### 3.2 Carbohydrates, Alcohols, and Organic Acids During Fermentation

**FIGURE 2:**
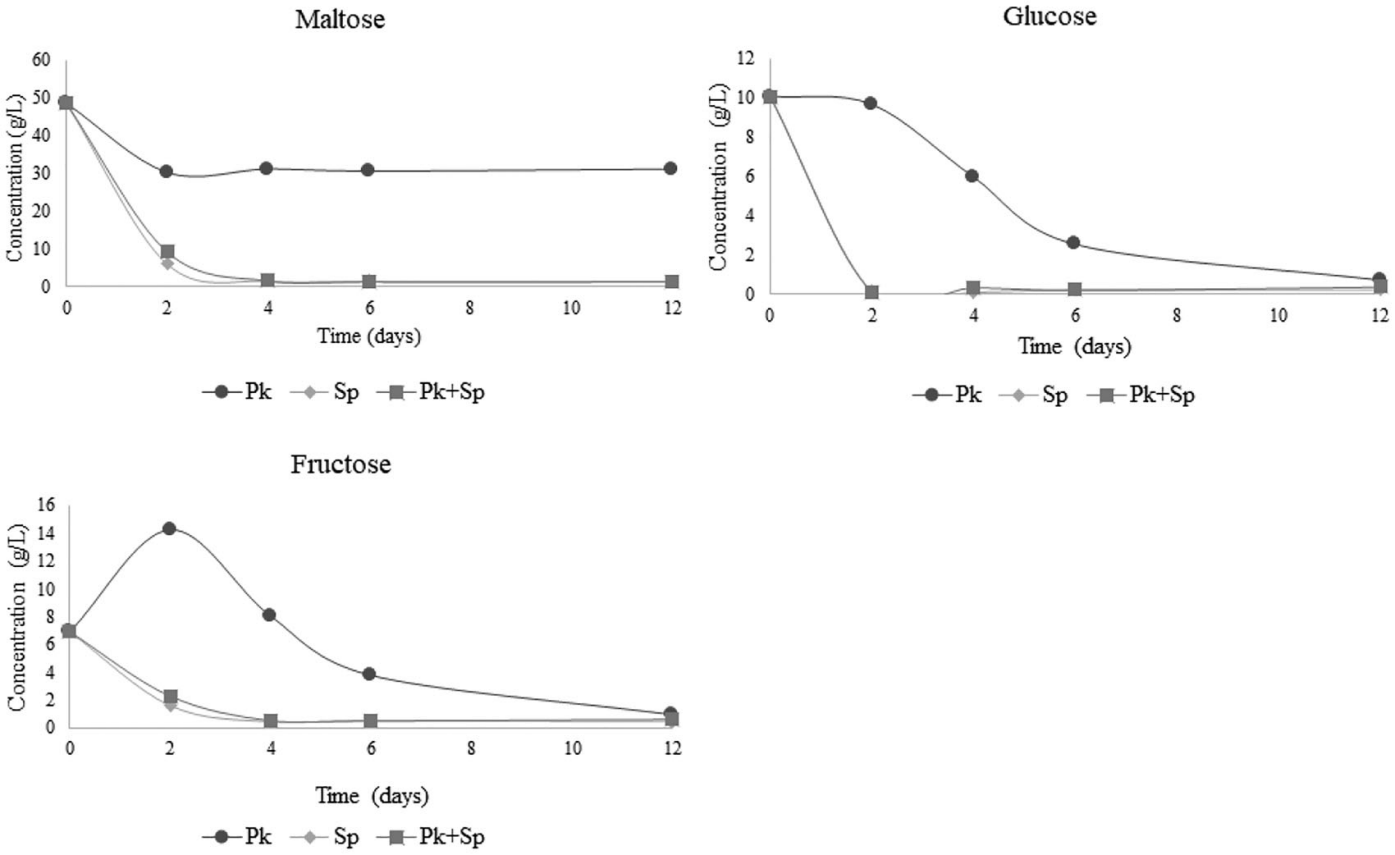
Concentration (g/L) of maltose (A), glucose (B), and fructose (C) during the 12 days of fermentation in the different trials using *P. kluyveri* L131 and commercial *S. pastorianus* in single and coculture. *P. kluyveri* L131 (Pk), commercial *S. pastorianus* (Sp), and *P. kluyveri* L131 plus commercial *S. pastorianus* (Pk+Sp).

The concentrations of maltose, glucose, and fructose were evaluated during the 12 days of fermentation and are presented in Figure 2. Maltose, glucose, and maltotriose are commonly found in beer wort (Zastrow et al. 2000). Adding fruit pulp such as umbu to beer wort also contributes to fermentable sugars, such as glucose and fructose, consumed by yeast during fermentation. The initial values of these carbohydrates in the beer wort with adding umbu pulp were 50.38 g/L for maltose, 11.21 g/L for glucose, and 6.05 g/L for fructose.

The present study showed that consuming the three carbohydrates (maltose, glucose, and fructose) was similar in the assays inoculated with *S. pastorianus* (single and co‐fermentation). In these assays, consumption occurred mainly in the first 4 days. However, the assay inoculated only with *P. kluyveri* L131 showed a later consumption of carbohydrates, corroborating the apparent extract (°P) data and CO_2_ losses. At 10 days of fermentation, the residual glucose values were similar among the three assays, while for fructose, the assay inoculated with a single culture of *P. kluyveri* L131 showed a higher (*p* < 0.05) residual concentration, 0.99 g/L, than the assays containing *S. pastorianus* (0.44 and 0.59 g/L for single culture and co‐fermentation, respectively).

Regarding maltose, a high concentration of 31.147 g/L was observed at the end of fermentation in the wort inoculated only with *P. kluyveri* L131, while the tests inoculated with *S. pastorianus* (single culture and cocultivation) showed concentrations lower than 1.5 g/L. It is important to emphasize that the *P. kluyveri* species has a low fermentation capacity, mainly in the metabolization of maltose, which justifies the low consumption of this carbohydrate and small ethanol production (6.07 g/L). Furthermore, an initial increase in fructose was observed during fermentation inoculated with a single culture of *P. kluyveri*. It may be associated with its invertase activity on the initially present sucrose in the wort, which releases glucose and fructose into the medium, combined with the greater affinity of yeasts for glucose. Thus, fructose can accumulate transiently, being consumed in later stages of the fermentation process. This was especially observed in fermentation performed with a single culture of *P. kluyveri*, which has a slower fermentation activity than Saccharomyces spp.

**FIGURE 3:**
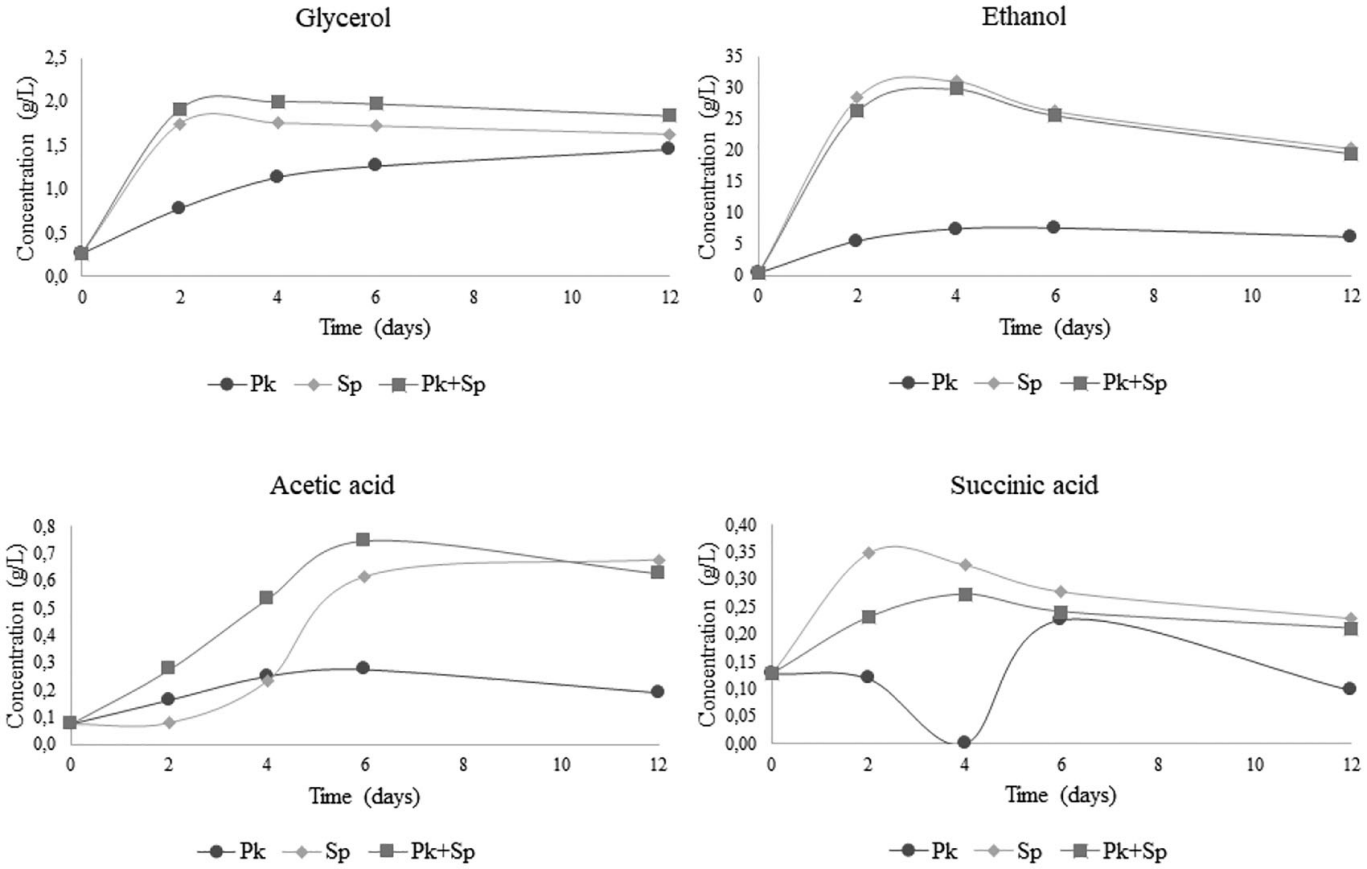
Concentrations (g/L) of glycerol (A), ethanol (B), acetic acid (C), and succinic acid (D) produced during the 12 days of fermentation in the different trials using *P. kluyveri* L131 and commercial *S. pastorianus* in single and cocultivation. *P. kluyveri* L131 (Pk), commercial *S. pastorianus* (Sp), and *P. kluyveri* L131 plus commercial *S. pastorianus* (Pk+Sp).

The concentrations of ethanol, glycerol, acetic, and succinic acids during fermentation are shown in Figure 3. Notably, ethanol production was lower in the assay with the single culture of *P. kluyveri* L131 throughout the fermentation, reaching a concentration of 6.07 g/L (0.8% v/v) (Figure 3B) at the end. The low ethanol production is probably related to the low fermentative capacity of the yeast, especially from maltose. The assays inoculated with *S. pastorianus* in single culture and coculture showed high ethanol production in the first 2 days, remaining constant until the end of fermentation and obtaining values of 20.32 g/L (2.6% v/v) and 19.51 g/L (2.5% v/v) for the single and co‐fermentation assays, respectively, at the end of 12 days. These results demonstrate that nonconventional yeasts such as *P. kluyveri* can be indicated to produce low‐alcohol beers, defined as greater than 0.5% and up to 2.0% v/v by Brazilian legislation and between 0.5% and 1.2% v/v in most European Union countries (Brasil 2019; Okaru and Lachenmeier 2022). In the United States, there are no official standards to define low alcohol beer, but nonalcoholic beer is defined as having a maximum of 0.5% v/v alcohol by volume (v/v) (Myles et al. 2022; Okaru and Lachenmeier 2022). Beers with reduced alcohol content have been emerging as consumer demand for beverage options with reduced alcohol profiles for healthy, cultural, and other reasons.

Glycerol is a metabolite resulting from the alcoholic fermentation of yeasts, influencing the sensory profile of the beer and impacting the perception of sweetness and viscosity of the final product (Mastanjević et al. 2018). The assays inoculated with *S. pastorianus* (single and coculture) showed faster production (in the first 2 days) than the assay containing single culture *P. kluyveri* (over 6 days). However, at the end of fermentation, there were no significant differences (*p* > 0.05) in glycerol concentrations (1.4–1.8 g/L) (Figure 3A).

Numerous studies (J.E. Rodrigues et al. 2010; Das et al. 2014; Kozaki et al. 2019) have demonstrated the presence of organic acids in beer, including malate, citrate, fumarate, lactate, succinate, acetate, and others. The content of these organic acids in beer exhibits a wide variation and impacts the perception of bitterness or salty notes (Das et al. 2014). The acid profile showed variations during the fermentation in the three trials (Figure 3C,E). At the end of the 12 days of fermentation, the trial using only the nonconventional yeast *P. kluyveri* L131 presented lower concentrations (*p* < 0.05) of acetic acid and succinic acid, 0.192 and 0.098 g/L, respectively, than the other trials containing *S. pastorianus* (single culture and co‐fermentation). These results highlight that the choice of yeast for beer production is of great importance, as it significantly impacts the chemical characteristics of the beer. The use of *P. kluyveri* L131 in a single culture resulted in a beer with lower alcohol, succinic acid, and acetic acid concentrations than beers produced with the commercial culture *S. pastorianus*. The co‐fermentation with commercial yeast and *P. kluyveri* L131 did not alter the ethanol, glycerol, and organic acid contents evaluated at the end of 12 days. However, as demonstrated in Figure 1, the fermentations inoculated with *S. pastorianus* were faster and could be completed in 4 days, a period normally used for beer production. At 4 days, it is evident that the concentration of succinic acid was lowest, while acetic acid was highest in the trial using co‐fermentation with both yeasts (Figure 3). These results indicate that the yeast used can influence the sensory characteristics of the beer. Lower succinic acid production is observed in *P. kluyveri* yeast cultures, especially during the first 4 days of fermentation. *P. kluyveri* tends to have a more respiratory metabolism than *Sacharromyces* spp. When oxygen is present, it consumes TCA intermediates instead of accumulating citrate. This may explain the reduced citric acid excretion in the early stages by *P. kluyveri*. Acetic acid significantly impacts beer's flavor, being perceived as harsh and contributing a sharp sourness and vinegary notes when its concentration exceeds the threshold of approximately 0.2 g/L (de Roos et al. 2018; 2020). In the present study, only the trial using a single culture of *P. kluyveri* obtained a concentration of acetic acid above the threshold value. While high levels of acetic acid can be problematic in non‐sour beers or when present in excessive amounts in sour beers, it is essential for achieving the distinct flavor profile and refreshing character typical of most sour beers (Witrick et al. 2020; de Roos et al. 2018; 2020; Bouchez and De Vuyst 2022).

### 3.3 Volatile Compounds Analysis

**FIGURE 4:**
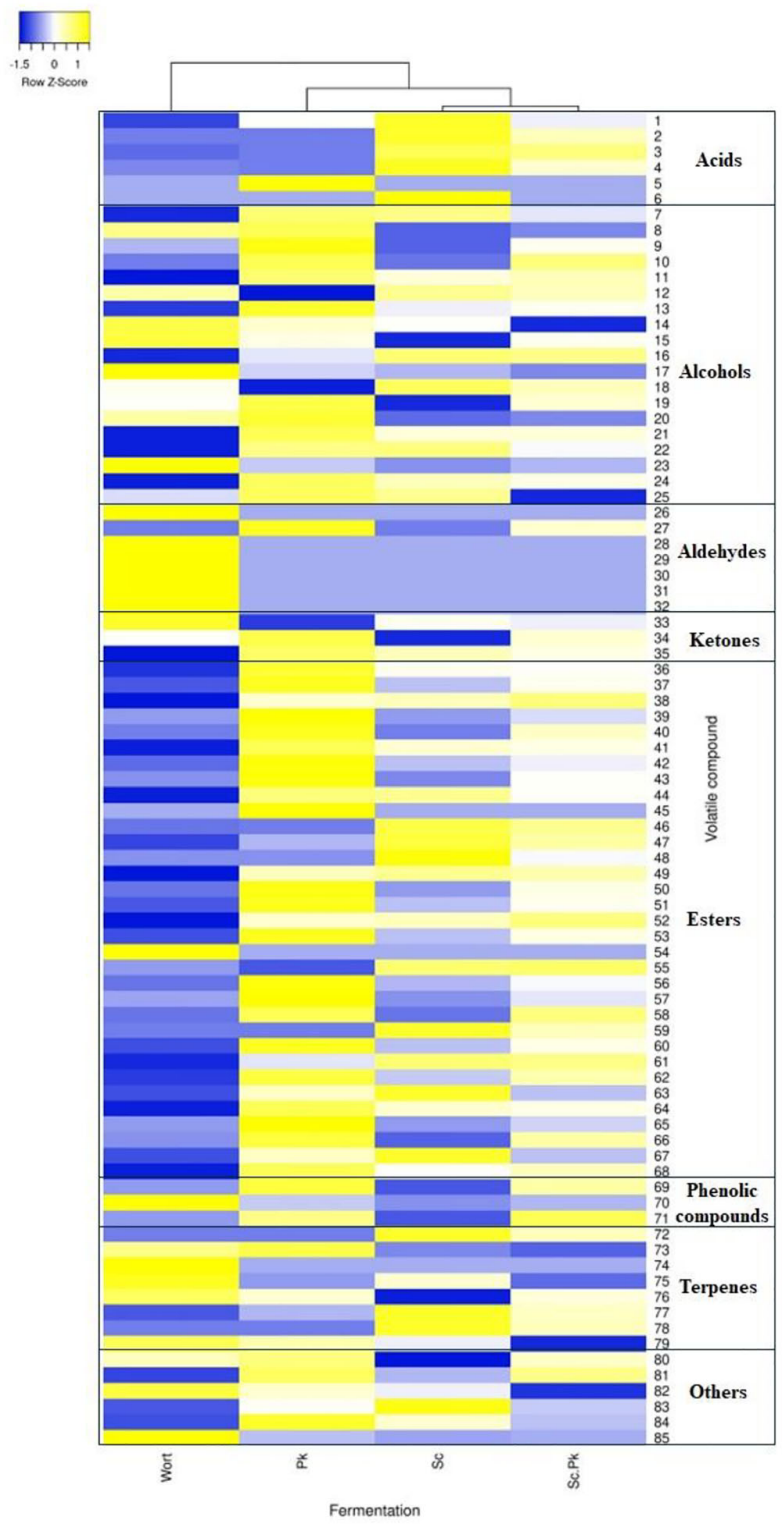
Heat maps of volatile compounds and their relative contents detected in brewing wort and fermented wort inoculated with *P. kluyveri* L131 (Pk), commercial *S. pastorianus* (Sp) and commercial *S. pastorianus* plus *P. kluyveri* (Sp + Pk).

The profile of volatile compounds was evaluated in the brewing wort and in the samples fermented with *P. kluyveri* L131 and commercial *S. pastorianus* in single and coculture at the end of fermentation. The results are shown in Figure 4 and Table S1.

A total of 85 volatile compounds were detected in all fermentation assays, including 33 esters, 19 alcohols, 8 terpenes, 7 aldehydes, 6 acids, 3 ketones, 3 phenolic compounds, and 6 others. According to the heatmap (Figure 4), the wort showed the most distant profile. While the assays inoculated with commercial *S. pastorianus* in single and cocultivation with *P. kluyveri* L31 were similar. The wort was highlighted by the presence of aldehydes, including benzaldehyde and 2,4‐dimethyl benzaldehyde, and terpenes, including camphene hydrate, caryophyllene, *cis*‐*p*‐mentha‐1(7),8‐dien‐2‐ol, and terpineol, in addition to the phenol compound. These compounds are commonly found in the hop and malt (Gribkova et al. 2023). On the other hand, the assay inoculated with the single culture *P. kluyveri* L131 demonstrated the presence of esters and alcohols, including ethyl‐9,12‐octadecadienoate, 3‐methylthiopropyl acetate, benzyl carbyl butyrate, geranyl acetate, 3‐phenyl‐1‐propanol, acetate, phenylethyl isovalerate, neryl acetate, phenethyl acetate, phenethyl isobutyrate, phenethyl propionate, phenylethyl alcohol, which are characterized by fruity and floral aromas (Pires et al. 2014; Bellut et al. 2018; Canonico et al. 2021). These compounds contribute to the complex aroma and flavor profiles of beer. Specifically, esters such as phenethyl acetate and geranyl acetate are associated with rose, honey, and fruity notes, which are highly appealing in specialty and craft beer markets. The sensory profile created by *P. kluyveri* is particularly relevant to consumers who prefer beers with pronounced aromatic character and complexity, such as fruit‐infused ales, Belgian‐style beers, or low‐alcohol and nonalcoholic options where yeast‐derived aroma compounds become more noticeable due to the absence of strong hop or malt flavors. The yeast *P. kluyveri* has been associated with ester production in different fermented foods and beverages, such as cocoa fermentation, wine, and beers, and has been evaluated as a starter culture due to its desired volatile compounds production (Crafack et al. 2013; Vicente et al. 2021; Methner et al. 2022; Piraine et al. 2023). This yeast can be considered a valuable tool for brewers aiming to tailor products to emerging consumer trends favoring more expressive and differentiated flavor profiles.

The assay inoculated with the single culture commercial *S. pastorianus* produced mainly acid compounds and some esters such as hexanoic acid, *n*‐decanoic acid, octanoic acid, ethyl caprate, ethyl dodecanoate, and ethyl‐ 9‐decenoate. Medium‐chain fatty acids and their ethyl esters can contribute to mouthfeel and background fruity notes (Pires et al. 2014). These compounds are part of the classical lager flavor matrix and appeal to consumers who prefer clean, crisp, and well‐attenuated beer styles (Bellut et al. 2018). Interestingly, some compounds commonly found in beer, such as ethyl acetate, acetaldehyde, and amyl alcohol, were not detected in this study. This may be due to the addition of umbu pulp, which led to an increase in the concentration of phenolics and simple carbohydrates such as glucose, potentially affecting yeast fermentation activity and the production of secondary compounds (Carvalho and Guido 2022; Langos and Granvogl 2016).

The co‐inoculation demonstrated that both yeasts could contribute to the volatile compound profile since most of the compounds found in the single cultures were also detected in the cocultivation. However, various compounds demonstrated lower concentrations in cocultivation than in single fermentation (Figure 4 and Table S1). The yeast can compete for substrate, decreasing the production of some secondary metabolites. This modulation may be advantageous or unfavorable depending on the intended sensory profile; for instance, it could attenuate excessively dominant ester notes, resulting in a more harmonized aroma composition, or alternatively, it may reduce the intensity of the fruity and floral attributes that are particularly desirable in specific market segments.

Therefore, *P. kluyveri* demonstrated a strong capacity to enhance the aromatic complexity of beer through the production of fruity and floral esters and higher alcohols. In contrast, *S. pastorianus* maintained its role as a conventional brewing yeast, producing medium‐chain fatty acids and their esters that contribute to the clean, balanced sensory profile characteristic of lager beers. Co‐inoculation revealed competitive interactions that modulated volatile and phenolic profiles, which may be strategically exploited to attenuate dominant notes or achieve greater sensory balance.

### 3.4 Antioxidant Activity and Phenolic Compounds

**FIGURE 5:**
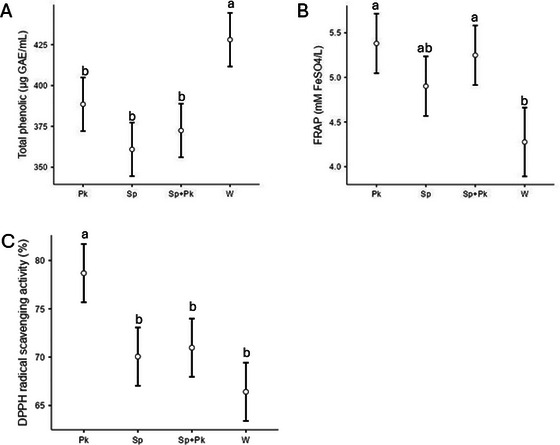
Total phenolic compounds (A) and antioxidant activity evaluated by FRAP (B) and DPPH radical scavenging activity (%) (C) of the brewing wort (W) and fermented wort inoculated with *P. kluyveri* L131 (Pk), commercial *S. pastorianus* (Sp) and commercial *S. pastorianus* plus *P. kluyveri* (Sp+Pk). The same letters in each graph indicate that the values did not differ according to the Tukey test at a probability of 5%.

The antioxidant activity and total phenolic compounds were evaluated, and the results are shown in Figure 5. The profile of phenolic compounds was also evaluated by HPLC analysis, and the results are shown in Table 1. The wort showed the highest total phenolic content (Figure 5A and Table 1) and the lower antioxidant activity assessed by FRAP and DPPH (Figure 5B,C), although it showed a similar EC50 to the sample fermented by *S. pastorianus* + *P. kluyveri*. According to Fumi et al. (2011), the fermentation process can reduce TPC as observed by tannins decrease, followed by flavonoids. Yeast cells have negatively charged cell walls associated with the ionization of carboxyl of cell wall proteins and phospho‐diester groups of phosphomannans (polysaccharides) that can bind phenolic compounds (Fumi et al. 2011; Stewart 2018; Carvalho and Guido 2022). It has been described that the mannan content in yeast cell walls can affect the adsorption of polyphenols (Harbah et al. 2020). Although the TPC in the beers produced by the different assays was similar, the amount of some phenolic compounds differed among the assays with different yeasts.

**TABLE 1:** Concentration (mg/L) of phenolic compounds detected in the brewing wort and fermented wort inoculated with single and coculture of *P. kluyveri* L131 and commercial *P. pastorianus*.

Compound (mg/L)	Wort	*P. kluyveri*	*S. pastorianus*	*P. kluyveri + S. pastorianus*
Caffeic acid	0.14 ± 0.003^a^	0.08 ± 0.02^c^	0.10 ± 0.001^b^	0.11 ± 0.01^b^
Sinapic acid + ferulic acid	0.12 ± 0.002^b^	0.14 ± 0.001^a^	0.13 ± 0.01^ab^	0.11 ± 0.02^b^
Gallic acid	2.82 ± 0.15^a^	2.12 ± 0.23^b^	2.54 ± 0.08^a^	2.26 ± 0.03^ab^
4‐hydroxybenzoic acid	ND	ND	ND	ND
*p*‐coumaric acid	0.14 ± 0.003^c^	0.20 ± 0.001 ^a^	0.18 ± 0.01^b^	0.17 ± 0.01^b^
5‐caffeoylquinic acid	0.20 ± 0.002^b^	0.22 ± 0.02^b^	0.27 ± 0.02^a^	0.23 ± 0.001^b^
Catechin	3.86 ± 0.035^a^	1.52 ± 0.09^b^	0.34 ± 0.01^c^	0.14 ± 0.001^c^
3,4‐dihydroxybenzoic acid	0.28 ± 0.006^a^	0.21 ± 0.001^b^	0.21 ± 0.01^b^	0.23 ± 0.05^ab^
Quercetin	ND	ND	ND	ND
Σ quantified phenolics	7.59 ± 0.12^a^	4.46 ± 0.31^b^	3.76 ± 0.08^c^	3.23 ± 0.11^d^

*Note*: Results were expressed as mean ± standard deviation of triplicates.

Different subscript letters in the same line indicate significant differences among samples. ANOVA two‐way followed by post‐test of Tukey at a probability of 5%.

Abbreviation: ND, not detected.

Using HPLC, which is recognized as a more sensitive and robust method for analyzing phenolic compounds in food matrices, we confirmed that the total phenolic content was significantly higher (*p* < 0.05) in wort (7.59 mg/L) compared to all fermented samples. This finding indicates a reduction in phenolic compounds during the fermentation process. Catechin and gallic acid were the predominant phenolic compounds in wort and fermented samples. However, gallic acid was prevalent in samples fermented with the combination of *P. kluyveri* and *S. pastorianus*, while catechin was not. Fermentation with *P. kluyveri* retained significantly more catechin (1.52 mg/L) compared to *S. pastorianus* (0.34 mg/L) and the combined yeasts (0.14 mg/L). Conversely, fermentation with *S. pastorianus* retained higher levels of gallic acid (2.54 mg/L) than *P. kluyveri* (2.12 mg/L). Caffeic, sinapic, ferulic, 5‐caffeoylquinic, and 3,4‐dihydroxybenzoic acids were less abundant in both wort and fermented samples. Notably, *P. kluyveri* fermentation yielded the highest levels (*p* < 0.05) of *p*‐coumaric acid (0.20 mg/L) and sinapic acid + ferulic acid (0.14 mg/L), although it had the lowest caffeic acid content. In contrast, *S. pastorianus* fermentation produced the highest concentration (*p* < 0.05) of 5‐CQA (0.27 mg/L). No significant differences (*p* > 0.05) were observed in the content of 3,4‐dihydroxybenzoic acid among the fermented samples.

In this study, we found that the process of transforming wort into beer resulted in a decrease in total phenolic compounds. This reduction was mainly attributed to a significant drop in catechin content (*p* < 0.05), which aligns with the findings of Viana et al. (2021). The reduction of specific phenolic compounds during fermentation can be attributed to the enzymatic activity of yeasts (Langos and Granvogl 2016), resulting in a distinct profile of phenolic compounds depending on the yeast strain used. Notably, *P. kluyveri* retained a higher total phenolic content than *S. pastorianus*, while the combination of both yeasts showed the lowest phenolic retention, suggesting potential competitive interactions affecting phenolic metabolism. These findings underscore the critical role of yeast strain selection in shaping beer's phenolic composition and antioxidant potential, with *P. kluyveri* demonstrating effectiveness in preserving certain phenolics.

Regarding the antioxidant activity, the assay inoculated with *P. kluyveri* showed the highest (*p* < 0.05) activity percentages by both methods, FRAP and DPPH. Studies have shown that *Pichia* species can enhance antioxidant activity in beer fermentation (Durga Prasad et al. 2022). Amongst the fermented samples, those inoculated with the single *P. kluyveri* culture showed the highest (*p* < 0.05) concentration of phenolic compounds identified, 4.46 mg/L (Table 1), which may have contributed to high antioxidant activity. FRAP analysis of iron‐reducing antioxidant power showed higher activity in fermented assays (Figure 5). These results indicate that antioxidant activity is not solely attributed to total phenolic content. This is evident from the wort with the highest phenolic concentration having the lowest antioxidant activity. The fermentation process can cause an increase in the antioxidant properties of beer (Durga Prasad et al. 2022) and other fermented beverages. This activity can also be influenced by other compounds such as melanoidins (formed during the Maillard reaction) (Martinez‐Gomez et al. 2020), vitamins B6, B12, E, and C, which, together with selenium, are recognized as micronutrients that increase the total antioxidant capacity of beers (Yang and Gao 2021). Additionally, the transformation of phenolics during fermentation also contributes to this increased activity (de Miranda et al. 2022).

Therefore, *P. kluyveri* was able to retain higher levels of phenolic compounds and antioxidant activity in the present study, highlighting its potential as a nonconventional starter culture for enhance functional propertie of the product.

### 3.5 Antimicrobial Activity

Antimicrobial activity of the beers was evaluated and is shown in Table 2. The beers produced by co‐fermentation of *P. kluyveri* and *S. pastorianus* showed the best results in terms of antimicrobial activity, with a MIC of 500 µL/mL, and were the only ones to show bactericidal activity against *L. monocytogenes*. Complementarily, *S. pastorianus* showed antimicrobial activity against *S. aureus* and *L. monocytogenes*, while *P. kluyveri* showed activity against enteropathogenic strains of *E. coli*. These results highlight the potential of co‐fermentation to eliminate foodborne pathogens, particularly *L. monocytogenes*, as it was the only study to show bactericidal activity.

**TABLE 2:** Antimicrobial activity of beers produced by different trials inoculated with *P. kluyveri* L131 (Pk), commercial *S. pastorianus* (Sp), and *P. kluyveri* L131 plus commercial *S. pastorianus* (Pk + Sp).

	Antimicrobial activity		
Strains	Pk	Sp	Pk+Sp
*S. aureus* ATCC13565	NA	500 MIC (µL/mL)	NA
*L. monocytogenes* CLIST2033	NA	500 MIC (µL/mL)	500 MIC and MBC (µL/mL)
*E. coli* enteropathogenic INCQS 00185	500 MIC (µL/mL)	NA	NA
*E. coli* O157:H7 RJ 691/1	NA	NA	NA
*Salmonella thyphimurium* IOC 190	NA	NA	NA
*Salmonella enteritidis* 8806/9	NA	NA	NA

Abbreviation: MBC, minimal bactericidal activity; MIC, minimal inhibitory concentration; NA, no activity was found.

Beer has previously demonstrated antimicrobial activity against *E. coli* and *Salmonella* spp. pathogens. This has been attributed to the synergistic effect of ethanol, organic acids, and hop‐derived iso‐α‐acids (Hatoum et al. 2012). The present study suggests that the antimicrobial effects observed in the co‐fermented beers may also be due to the unique interaction of the yeast strains. Fermentations involving different yeast strains or even cocultures have been shown to produce diverse antimicrobial compounds, especially from non‐*Saccharomyces* yeasts (Fernandes et al. 2021; Kayadelen et al. 2023).

Investigation of the contribution of ethanol to the observed effects revealed that ethanolic solutions at the same concentrations (% v/v) as the samples, as well as the initial wort, showed no antimicrobial activity. These results suggest that neither ethanol nor the initial fermentation medium is responsible for the observed antimicrobial effects. Furthermore, no antimicrobial activity was observed for the pH‐neutralized beer samples, suggesting that organic acids can play a central role. In their undissociated form (HA), organic acids exhibit lipophilic properties that enable them to penetrate the lipid bilayer of microbial cell membranes. Following neutralization, the acids predominantly exist in their dissociated form (A^−^), which is polar and thus unable to permeate across the plasma membrane. This shift in chemical form results in a loss of antimicrobial activity, despite the total acid concentration remaining constant (Dysvik et al. 2020). Studies have shown that fermentation can generate antimicrobial metabolites such as organic acids, higher alcohols, and phenolic compounds capable of inhibiting pathogenic microorganisms (Fernandes et al. 2021; Çobo et al. 2023; Kayadelen et al. 2023).

While traditional literature links alcohol content of 3.5% to 5% in conventional beers with pathogen inhibition (Çobo et al. 2023), this study suggests that in nonconventional beers with lower alcohol content (0.8% to 2.6%), the observed antimicrobial activity is more likely due to specific bioactive compounds, such as organic acids, produced during fermentation.

This study advances over years of brewing research by expanding the scientific framework of beer production beyond traditional barley/hop/*Saccharomyces* systems to include a native tropical fruit (umbu, *S. tuberosa*) and nonconventional yeast strains. By systematically evaluating fermentation performance and technological feasibility using both conventional and nonconventional yeasts, the work demonstrates that umbu is not merely a flavoring adjunct but an active fermentation matrix that modulates yeast metabolism, acid balance, and aroma development. The comparative fermentation approach provides new insights into yeast and substrate interactions in fruit‐based beers, a topic that remains underexplored in brewing science relative to wine fermentation. From a technological perspective, the study confirms the compatibility of umbu with standard brewing processes while highlighting the potential of non‐*Saccharomyces* yeasts as tools for product differentiation and sensory innovation. Collectively, these findings contributed to the field of food and fermentation science by validating the use of underutilized regional biodiversity in beer production, thereby supporting the diversification of raw materials and yeast resources within modern brewing technology.

## 4 Conclusion

The results demonstrate that the choice of yeast and fermentation strategies significantly affects the chemical composition and functional properties of beer. *Pichia kluyveri* L131 showed potential for producing low‐alcohol beers with enhanced antioxidant activity and distinct aromatic profiles. The fermentation by this nonconventional yeast generates beers with diverse volatile compounds, mainly esters, and retained phenolics, showing high antioxidant activity. Meanwhile, *S. pastorianus* offers efficient sugar metabolism and rapid fermentation that are suitable for traditional beer production. cocultivation provided a balance between volatile compounds and fermentation efficiency. Furthermore, beers fermented with different yeast strains and cocultivation exhibited variable antimicrobial activities, emphasizing the role of yeast‐derived metabolites in inhibiting pathogens. This study demonstrates the potential of using unconventional yeast to produce beer, along with the addition of umbu pulp in the wort. Future research should investigate scalability and consumer acceptance to promote the industrialization and commercialization of innovative beers.

## Author Contributions

**Diego Pádua de Almeida**: investigation, writing – original draft, formal analysis, data curation. H**ygor Lendell Silva de Souza**: investigation, formal analysis, writing – original draft, data curation. **Ana Luiza Dozol**: formal analysis, investigation. **Mariana Moysés Delorme**: investigation, formal analysis. **Vanessa Naciuk Castelo‐branco**: investigation, formal analysis, writing – review and editing, data curation. **Adriene Ribeiro Lima**: investigation, writing – review and editing, formal analysis, data curation. **Alexandre Soares dos Santos**: investigation, formal analysis, data curation. **Nadia Nara Batista**: investigation, formal analysis, data curation. **Rosane Freitas Schwan**: writing – review and editing, investigation, methodology. **Gustavo Molina**: investigation, data curation, writing – review and editing, methodology. **Cintia Lacerda Ramos**: writing – review and editing, conceptualization, funding acquisition, data curation, supervision, investigation.

## Funding

This work was supported by Coordenação de Aperfeiçoamento de Pessoal de Nível Superior—Brazil (CAPES)—Finance Code 001, Fundação de Amparo à Pesquisa do Estado de Minas Gerais (Fapemig) grant number APQ‐01951‐21, APQ‐03106‐21 and APQ 4509‐23, and Conselho Nacional de Desenvolvimento Científico e Tecnológico do Brasil (CNPq) grant number 408307/2021‐8.

## Conflicts of Interest

The authors declare no conflicts of interest.

## Supporting information




**Supplementary Tables**: jfds70875‐sup‐0001‐Tables.docx

## Data Availability

Data will be available on request from the authors.
