# Supplementary material for: Beer Production With Umbu (Spondias tuberosa) Using Conventional and Nonconventional Yeast
Source: J Food Sci. 2026 Jan 18;91(1):e70875. doi: 10.1111/1750-3841.70875 (PMC12813584; doi:10.1111/1750-3841.70875)
Supplement: Supplementary file 1 — Supplementary Tables: jfds70875‐sup‐0001‐Tables.docx [file JFDS-91-0-s001.docx]

Supplementary Table 1. Volatile compounds (peak area x 10^4^) detected in the wort and after 264 h of fermentation produced in the assays inoculated with *P. kluyveri* L131, commercial yeast (Sc), and the co-culture of commercial yeast and *P. kluyveri* L131 (Sc + Pk).

| **ID** | **Compound** | Peak area (x10^4^) | | | |
| --- | --- | --- | --- | --- | --- |
|  |  | **Wort** | **Pk** | **Sc** | **Sc+Pk** |
|  | *Acids* |  |  |  |  |
| 1 | Dodecanoic acid | - | 1.2 | 2.5 | 1.1 |
| 2 | Hexanoic acid | - | - | 17.2 | 10.1 |
| 3 | n-Decanoic acid | 1.4 | 2.5 | 14.5 | 13.1 |
| 4 | Octanoic acid | 3.4 | 2.8 | 46.0 | 24.4 |
| 5 | Isobutyric acid | - | 7.1 | - | - |
|  | *Alcane* |  |  |  |  |
| 6 | n-Octadecane | - | - | 20.2 | - |
|  | *Alcohois* |  |  |  |  |
| 7 | 1-Dodecanol | 1.4 | 6.4 | 6.1 | 4.1 |
| 8 | 2-Ethyl-1-hexanol | 3.8 | 41.8 | 15.1 | 18.3 |
| 9 | 1-Nonanol | 1.3 | 6.1 | - | 2.7 |
| 10 | 1-Octanol | 20.5 | 35.0 | 19.9 | 33.2 |
| 11 | 2,6,10-Dodecatrien-1-ol, 3,7,11-trimethyl | - | 1.1 | 0.8 | 0.9 |
| 12 | 2-Furanmethanol | 21.9 | 9.6 | 22.6 | 21.0 |
| 13 | 2-Methyl-1-undecanol | 2.2 | 7.0 | 4.5 | 4.8 |
| 14 | (R)-(-)-Menthol | 14.5 | 12.9 | 12.4 | 9.6 |
| 15 | 2-Decen-1-ol | 2.4 | 1.4 | - | 1.4 |
| 16 | 1-Decanol | 5.6 | 15.8 | 24.3 | 23.3 |
| 17 | Fenchyl alcohol | 33.8 | 13.5 | 11.4 | 8.2 |
| 18 | Furfuryl alcohol | 20.9 | 9.6 | 27.1 | 23.2 |
| 19 | Geraniol | 32.5 | 37.3 | 26.0 | 33.5 |
| 20 | 2-Ethyl-1-hexanol | 37.8 | 48.4 | 15.1 | 18.3 |
| 21 | 3,6-Nonadien-1-ol | 1.2 | 3.0 | 2.4 | 2.4 |
| 22 | Phenylethyl alcohol | 30.8 | 304.2 | 311.1 | 206.6 |
| 23 | Phenyl alcohol | 19.5 | 4.1 | 0.9 | 2.9 |
| 24 | Nerolidol | - | 1.3 | 1.0 | 0.8 |
| 25 | Linalool, formate | 114.9 | 130.0 | 125.7 | 99.6 |
|  | *Aldehyde* |  |  |  |  |
| 26 | 1,4-Benzenedicarboxaldehyde | 5.2 | - | - | - |
| 27 | 2-Acetoxytetralin | - | 1.7 | - | 0.8 |
| 28 | Benzaldehyde | 7.1 | - | - | - |
| 29 | 2,4-Dimethylbenzaldehyde | 5.2 | - | - | - |
| 30 | α-Ethylidenbenzeneacetaldehyde | 1.5 | - | - | - |
| 31 | 2-Phenyl-2-butenal | 1.5 | - | - | - |
| 32 | 5-Methyl-2-phenyl-2-hexenal | 0.9 | - | - | - |
|  | *Ketones* |  |  |  |  |
| 33 | 2-Buten-1-one, 1-(2,6,6-trimethyl-1,3-cyclohexadien-1-yl)-, (E)- | 6.7 | - | 3.5 | 3.0 |
| 34 | 5,9-Undecadien-2-one, 6,10-dimethyl-, (Z)- | 32.5 | 37.3 | 26.0 | 33.5 |
| 35 | Ethanone, 1-(2-furanyl)- | - | 24.5 | 19.9 | 17.4 |
|  | *Esters* |  |  |  |  |
| 36 | Diisobutyl phthalate | 43.3 | 117.7 | 84.2 | 82.9 |
| 37 | 2-trans-6-trans-Farnesyl acetate | - | 4.1 | 1.2 | 2.0 |
| 38 | Furfuryl acetate | - | 43.2 | 46.2 | 54.7 |
| 39 | 3-Methylthiopropyl acetate | - | 33.0 | - | 6.0 |
| 40 | Ethyl-9,12-octadecadienoate | - | 0.5 | - | 0.3 |
| 41 | Ethyl-9,12-octadecadienoate | 896.6 | 4482.9 | 3369.6 | 3159.3 |
| 42 | Octyl acetate | - | 8.6 | 2.1 | 3.1 |
| 43 | Benzyl carbyl butyrate | 2.0 | 26.7 | 1.1 | 10.3 |
| 44 | cis-3-Hexen-1-yl butyrate | - | 1.9 | 1.8 | 1.3 |
| 45 | Phenethyl 2-methylbutyrate | - | 4.9 | - | - |
| 46 | Ethyl caprate | 57.6 | 83.1 | 815.6 | 639.1 |
| 47 | [Ethyl dodecanoate](https://www.ncbi.nlm.nih.gov/pcsubstance/?term=%22Ethyl%20dodecanoate%22%5bCompleteSynonym%5d%20AND%207800%5bStandardizedCID%5d) | 7.8 | 19.3 | 43.7 | 34.6 |
| 48 | Ethyl 9-decenoate | - | - | 44.4 | 14.4 |
| 49 | Ethyl 9-hexadecenoate | 0.9 | 3.0 | 3.3 | 3.1 |
| 50 | Ethyl Oleate | 0.2 | 1.3 | 0.3 | 0.7 |
| 51 | Farnesyl acetate | - | 4.1 | 1.2 | 2.0 |
| 52 | Furfuryl acetate | - | 43.2 | 46.2 | 54.7 |
| 53 | Geranyl acetate | 3.7 | 89.6 | 29.3 | 49.1 |
| 54 | Ethyl 3-hydroxyhexanoate | 1.6 | - | - | - |
| 55 | Ethyl hydrocinnamate | 3.6 | - | 16.2 | 17.0 |
| 56 | 3-Phenyl-1-propanol, acetate | - | 6.6 | 1.2 | 2.4 |
| 57 | Phenylethyl isovalerate | 8.4 | 114.4 | 2.2 | 31.5 |
| 58 | Ethyl linoleate | - | 0.7 | - | 0.6 |
| 59 | iso-Butyl caprylate | - | - | 3.5 | 2.0 |
| 60 | Neryl acetate | 3.7 | 89.6 | 29.3 | 49.1 |
| 61 | Ethyl nonanoate | - | 8.3 | 14.7 | 14.0 |
| 62 | Ethyl palmitate | 1.4 | 5.4 | 2.9 | 4.3 |
| 63 | Pentanoic acid, 2,2,4-trimethyl-3-carboxyisopropyl, isobutyl ester | - | 17.8 | 28.8 | 8.6 |
| 64 | Phenethyl acetate | 896.6 | 4482.9 | 3369.6 | 3159.3 |
| 66 | Phenethyl isobutyrate | - | 25.9 | - | 4.3 |
| 66 | Phenethyl propionate | 3.6 | 23.8 | - | 17.0 |
| 67 | 3-hydroxy-2,2,4-trimethylpentyl isobutyrate | 0.5 | 2.5 | 3.8 | 1.5 |
| 68 | Ethyl tetradecanoate | 0.9 | 3.0 | 2.2 | 2.4 |
|  | *Phenolic compounds* |  |  |  |  |
| 69 | 4-Ethylguaiacol | 1.4 | 3.4 | 0.9 | 2.7 |
| 70 | Phenol | 19.5 | 4.1 | 0.9 | 2.9 |
| 71 | Phenol, 2,4-bis-(1,1-dimethylethyl), TMS | 5.7 | 7.1 | 5.2 | 7.3 |
|  | *Terpenes* |  |  |  |  |
| 72 | [2,6-dimethyl-2,6-octadiene](https://webbook.nist.gov/cgi/cbook.cgi?ID=T999907823) | - | - | 10.4 | 6.1 |
| 73 | Delta cadinene | 2.6 | 2.9 | 1.4 | 1.2 |
| 74 | Camphene hydrate | 3.9 | - | - | - |
| 75 | Caryophyllene | 4.1 | 2.5 | 3.3 | 2.3 |
| 76 | cis-p-mentha-1(7),8-dien-2-ol | 2.4 | 2.0 | 0.9 | 2.0 |
| 77 | Citronellol | 3.8 | 10.5 | 30.1 | 19.5 |
| 78 | Citronellyl acetate | - | - | 10.4 | 6.1 |
| 79 | Terpineol | 128.8 | 113.7 | 98.3 | 64.8 |
|  | *Others* |  |  |  |  |
| 80 | Tetradecamethylcycloheptasiloxane | 8.8 | 10.0 | 2.9 | 8.5 |
| 81 | Dodecamethylcyclohexasiloxane | - | 3.2 | 1.1 | 2.8 |
| 82 | Dibutyl phthalate | 12.2 | 10.4 | 9.6 | 6.9 |
| 83 | Dibutylformamide | - | 4.0 | 8.9 | 2.5 |
| 84 | Homosalate | 0.5 | 1.4 | 1.0 | 0.8 |
| 85 | 2-Acetylpyrrole | 5.0 | 1.9 | 1.6 | 1.7 |

Supplementary Table 2. Parameters obtained in the wort and after 264 h of fermentation produced in the assays inoculated with *P. kluyveri* L131, commercial yeast (Sc), and the co-culture of commercial yeast and *P. kluyveri* L131 (Sc + Pk).

|  | **Fermentation assays** | | | | |
| --- | --- | --- | --- | --- | --- |
| **Parameters** | **Wort** | **Pk** | **Sc** | **Sc+Pk** |  |
| Alcohol (%) | 0.00±0.00 | 0.8±0.03 | 2.6±0.21 | 2.5±0.21 |  |
| Ethanol (g/L) | 0.37±0.01 | 6.07±0.21 | 20.32±1.66 | 19.51±1.61 |  |
| Glycerol (g/L) | 0.26±0.01 | 1.45±0.16 | 1.63±0.07 | 1.84±0.01 |  |
| Acetic acid (g/L) | 0.08±0.00 | 0.19±0.02 | 0.68±0.02 | 0.63±0.11 |  |
| DPPH (%) | 66.41±4.63 | 78.69±1.54 | 70.05±2.31 | 70.97±2.92 |  |
| FRAP (mM FeSO_4_/L) | 4.28 ±0.10 | 5.38±0.30 | 4.90±0.08 | 5.25±0.40 |  |
| Total phenolic (µg GAE/mL) | 428.06±10.67 | 388.51±8.23 | 360.89±5.04 | 372.46±26.45 |  |
| Σ Quantified phenolics (mg/L) | 7.59±0.12 | 4.46±0.31 | 3.76±0.08 | 3.23±0.11 |  |
